# The *Capsicum annuum* class IV chitinase ChitIV interacts with receptor-like cytoplasmic protein kinase PIK1 to accelerate PIK1-triggered cell death and defence responses

**DOI:** 10.1093/jxb/erv001

**Published:** 2015-02-17

**Authors:** Dae Sung Kim, Nak Hyun Kim, Byung Kook Hwang

**Affiliations:** Laboratory of Molecular Plant Pathology, College of Life Sciences and Biotechnology, Korea University, Seoul 136-713, Republic of Korea

**Keywords:** Cell death, class IV chitinase, defence, pepper, *Xanthomonas campestris* pv. *vesicatoria*.

## Abstract

The pepper receptor-like cytoplasmic protein kinase, CaPIK1, which mediates signalling of plant cell death and defence responses was previously identified. Here, the identification of a class IV chitinase, CaChitIV, from pepper plants (*Capsicum annuum*), which interacts with CaPIK1 and promotes CaPIK1-triggered cell death and defence responses, is reported. CaChitIV contains a signal peptide, chitin-binding domain, and glycol hydrolase domain. *CaChitIV* expression was up-regulated by *Xanthomonas campestris* pv. *vesicatoria* (*Xcv*) infection. Notably, avirulent *Xcv* infection rapidly induced *CaChitIV* expression in pepper leaves. Bimolecular fluorescence complementation and co-immunoprecipitation revealed that CaPIK1 interacts with CaChitIV *in planta*, and that the CaPIK1–CaChitIV complex is localized mainly in the cytoplasm and plasma membrane. CaChitIV is also localized in the endoplasmic reticulum. Transient co-expression of *CaChitIV* with *CaPIK1* enhanced *CaPIK1*-triggered cell death response and reactive oxygen species (ROS) and nitric oxide (NO) bursts. Co-silencing of both *CaChitIV* and *CaPIK1* in pepper plants conferred enhanced susceptibility to *Xcv* infection, which was accompanied by a reduced induction of cell death response, ROS and NO bursts, and defence response genes. Ectopic expression of *CaPIK1* in *Arabidopsis* enhanced basal resistance to *Hyaloperonospora arabidopsidis* infection. Together, the results suggest that CaChitIV positively regulates CaPIK1-triggered cell death and defence responses through its interaction with CaPIK1.

## Highlight

The pepper class IV chitinase CaChitIV interacts with the pepper receptor-like cytoplasmic protein kinase CaPIK1 and promotes CaPIK1-triggered cell death and defence responses. CaChitIV is localized to the endoplasmic reticulum.

## Introduction

Plants are exposed to a constant and diverse array of potential microbial pathogens and have developed the ability to protect themselves from pathogen attack by the early detection of disease-causing agents (Kenrick and Crane, 1997; Jones and Dangl, 2006). Recognition of microbial pathogens activates defence responses, including activation of mitogen-activated protein kinase (MAPK) cascades and accumulation of reactive oxygen species (ROS) and nitric oxide (NO), and activation of transcriptional factors, leading to the timely expression of pathogenesis-related (PR) genes (Chisholm *et al.*, 2006; van Loon *et al.*, 2006; Asai *et al.*, 2008; Kim and Hwang, 2011; Choi *et al.*, 2012; Meng and Zhang, 2013). The hypersensitive response (HR) is the most effective and best known plant response to pathogen attacks. It is a form of programmed cell death (PCD) in which cells around the infection site undergo rapid necrosis. The HR is associated with a co-ordinated and integrated set of metabolic modifications that are integral to hindering the further progress of pathogens, as well as to enhancing the ability of the host to limit subsequent infection by various pathogens (Greenberg, 1997; van Loon and Strien, 1999; Greenberg and Yao, 2004). The HR is activated by intracellular resistance (R) proteins which recognize effector proteins derived from avirulent pathogens, so-called effector-triggered immunity (ETI). ETI is generally characterized by the induction of HR at the site of infection and of systemic acquired resistance (SAR) at distal sites (Dangl and Jones, 2001; Chisholm *et al.*, 2006; Yao and Greenberg, 2006).

Protein kinases are well-characterized, essential proteins that act through phosphorylation as diverse key enzymes in signal transduction (Stone and Walker, 1995). A growing body of evidence highlights the importance of protein kinases in various aspects of plant immunity (Dardick *et al.*, 2007; Meng and Zhang, 2013). Plant receptor-like cytoplasmic protein kinases (RLCKs) belong to the superfamily of receptor-like kinases (RLKs). Well-known RLCKs include PBS1, PBL1, and BIK1 from *Arabidopsis thaliana* (L.) Heynh, and Pto, Pti, and Tpk1b from tomato (*Solanum lycopersicum* L.), which regulate plant immunity against biotrophic and necrotrophic pathogens (Martin *et al.*, 1993; Zhou *et al.*, 1995; Swiderski and Innes, 2001; AbuQamar *et al.*, 2008; Zhang *et al.*, 2010). In a previous study (Kim and Hwang, 2011), the pepper receptor-like cytoplasmic protein kinase, CaPIK1, which mediates signalling of cell death and defence responses to microbial pathogens was identified. *CaPIK1* expression in pepper plants (*Capsicum annuum* L.) triggers immune responses including ROS and NO bursts, as well as callose deposition, ultimately leading to HR-like cell death.

Plants produce many types of chitinases, which catalyse the degradation of chitin, a linear polymer of *N*-acetyl-d-glucosamine (GlcNAc). Chitinases are grouped into seven classes based upon their primary structure (Collinge *et al.*, 1993; Neuhaus *et al.*, 1996; Gomez *et al.*, 2002; Wiweger *et al.*, 2003). Different chitinase classes are defined depending on sequence similarities and the presence of an N-terminal cysteine-rich domain, usually referred to as hevein-like domain or chitin-binding domain (CBD), which is separated from the catalytic domain by a hinge region (Gomez *et al.*, 2002). Only chitinases of classes I and IV possess a CBD. Chitinases in class IV are phylogenetically related to class I and II chitinases (Gomez *et al.*, 2002; Wiweger *et al.*, 2003). Collinge *et al.* (1993) proposed that class IV chitinases evolved from class I through a series of four deletions, one of which removed a vacuole-targeting sequence; as a result, class IV chitinases are secreted to the apoplast rather than targeted to vacuoles.

It is known that plant chitinases play important roles in defence against pathogenic attacks (Gomez *et al.*, 2002; Hong and Hwang, 2002; Hietala *et al.*, 2004) and stress response (Hong and Hwang, 2006; Takenaka *et al.*, 2009), and in growth and development (Wiweger *et al.*, 2003). Many chitinases have been classified as pathogenesis-related proteins of the PR-3, PR-4, PR-8, and PR-11 families (Neuhaus *et al.*, 1996). Antifungal activity has been reported for chitinases that contain an N-terminal CBD (classes I and IV) and also for enzymes that lack such a domain (Schlumbaum *et al.*, 1986; Gomez *et al.*, 2002). There is increasing evidence that transgenic plants constitutively overexpressing chitinases exhibit elevated resistance to pathogens (Broglie *et al.*, 1991; Grison *et al.*, 1996; Shin *et al.*, 2008). Plant class IV chitinases are mainly involved in regulating resistance to fungal pathogens (Hietala *et al.*, 2004). There is some evidence that class IV chitinases are implicated in other processes, such as the response to abiotic stress (Gerhardt *et al.*, 2004) and defence against bacterial pathogens (Gerhardt *et al.*, 1997). However, our knowledge of the cellular mechanisms by which class IV chitinases activate plant cell death and innate immunity is still limited, and functional studies of class IV chitinases are needed to provide evidence for their distinct functions.

Previously, the pathogen-induced *CaPIK1* (pepper receptor-like cytoplasmic protein kinase) was identified as a positive regulator of plant cell death and defence responses (Kim and Hwang, 2011). In the current study, the pepper class IV chitinase, CaChitIV, which interacts with CaPIK1 in yeast and *in planta*, was isolated and functionally characterized Bimolecular fluorescence complementation (BiFC) and co-immunoprecipitation (Co-IP) experiments revealed that CaPIK1 interacts with CaChitIV *in planta*, with the CaChitIV–CaPIK1 complex being localized mainly to the cytoplasm and plasma membrane. CaChitIV is secreted to the apoplastic region via the endoplasmic reticulum (ER). Transient co-expression of *CaChitIV* with *CaPIK1* enhanced the *CaPIK1*-triggered cell death response and ROS burst, as well as NO burst. Virus-induced gene silencing (VIGS) of *CaChitIV* or/and *CaPIK1* in pepper plants conferred enhanced susceptibility to *Xanthomonas campestris* pv. *vesicatoria* (*Xcv*) infection. In contrast, heterologous *CaChitIV* overexpression in *Arabidopsis* enhanced basal resistance to *Hyaloperonospora arabidopsidis* (*Hpa*) infection. The results suggest that CaChitIV positively regulates ROS and NO burst, leading to plant cell death and defence responses through its interaction with CaPIK1.

## Materials and methods

### Plant growth and pathology assays

Pepper (*Capsicum annum* L., cv Nockwang) and tobacco (*Nicotiana benthamiana*) plants were grown in soil mix (peat moss/perlite/vermiculite, 2:1:1, v/v/v) at 26 °C with a photoperiod of 16h at a light intensity of 130 μmo lm^–2^ s^–1^ and 60% relative humidity in an environmental growth room.

*Arabidopsis thaliana* wild-type (ecotype Columbia, Col-0) and transgenic seeds were surface-sterilized with ethanol and washed, before undergoing imbibition at 4 °C for 3 d to overcome dormancy. Plants were grown in soil mix at 24 °C under long-day conditions (16h light/8h dark cycle) or under short-day conditions (12h light/12h dark) at a light intensity of 130 μmol m^–2^ s^–1^ and 60% relative humidity in an environmental growth chamber.

Virulent Ds1 and avirulent Bv5-4a strains of *Xcv* (Kim *et al.*, 2010) were cultured overnight in yeast nutrient broth (5g l^–1^ yeast extract, 8g l^–1^ nutrient broth), harvested, re-suspended in sterile tap water to a concentration of 5×10^4^ cfu ml^–1^, and used to infiltrate fully expanded pepper leaves. To inoculate *Arabidopsis* leaves, *Pseudomonas syringae* pv. *tomato* (*Pst*) DC3000 and DC3000 (*avrRpm1*) were grown in King’s B broth (10g l^–1^ peptone, 1.5g l^–1^ K_2_HPO_4_, 15g l^–1^ glycerol, and 5g l^–1^ MgSO_4_). Bacterial cultures were diluted to the appropriate density and infiltrated into plant leaves. The infected leaves were sampled at various time points for bacterial growth assay, RNA isolation, and histochemical assay.

Spores of *Hpa* isolate Noco2, known to be virulent to *Arabidopsis* ecotype Col-0, were collected in sterile tap water containing 0.05% Tween-20 from infected cotyledons and leaves. Spore suspensions (5×10^4^ conidiospores ml^–1^) were sprayed onto 7-day-old *Arabidopsis* seedlings, infected plants were covered with plastic wrap to maintain moisture, and the number of sporangiophores on cotyledons was counted to assess disease severity 7 d after infection. Infected cotyledons were sampled for histochemical assay after 3 d.

### Yeast two-hybrid screening

Yeast two-hybrid screening was conducted using the GAL4 system, according to the manufacturer’s instructions (Matchmaker™ GAL4 Two-Hybrid System 3, Clontech, CA, USA). The full-length *CaPIK1* coding regions were amplified using PCR and cloned into the *Eco*RI/*Bam*HI restriction sites of the bait vector pGBKT7, which includes the GAL4 DNA-binding domain (BD). A yeast two-hybrid cDNA library was constructed in the prey vector pGADT7, which contains a GAL4 activation domain (AD), using cDNA constructed from pepper leaves infected with the *Xcv* avirulent strain Bv5-4a.

Constructs were introduced into yeast strain AH109 using the lithium acetate-mediated transformation method, and transformants were arrayed on interaction selection media [SD-Adenine (Ade)-Histidine (His)-Leucine (Leu)-Tryptophan (Trp)], supplemented with 40mg l^–1^ 5-bromo-4-chloro-3-indoyl-α-d-galactoside (X-α-Gal), to score growth and colony colour as indicators of protein–protein interactions.

### Bimolecular fluorescence complementation (BiFC) analysis

BiFC analyses were conducted as described previously (Walter *et al.*, 2004). For the BiFC constructs, cDNAs encoding CaPIK1 and CaChitIV without termination codons were amplified using PCR and recombined into the binary vectors pSPYNE and pSPYCE, harboring YFP^N^ and YFP^C^ (yellow fluorescent protein N- and C-termini), respectively, under the control of the *Cauliflower mosaic virus* (CaMV) 35S promoter, resulting in CaPIK1-YFP^N^ and CaChitIV-YFP^C^. *Agrobacterium tumefaciens* strain GV3101 was transformed with the BiFC constructs, and cultures were co-infiltrated into *N. benthamiana* leaves. Three days after infiltration with *Agrobacterium*, leaves were visualized using an LSM5 Exciter confocal laser-scanning microscope (Carl Zeiss, Germany) with excitation at 514nm and emission at 525–600nm.

### Green fluorescent protein (GFP) fluorescence microscopy

For GFP constructs, the *CaChitIV* coding region and the signal peptide-deleted *CaChitIV* (*CaChitIVΔSP*) were PCR amplified and introduced into *Xba*I/*Bam*HI sites of the binary vector pBIN35S::326-GFP to generate a C-terminal soluble-modified GFP (smGFP)-tagged fusion protein. For particle bombardment, onion (*Allium cepa* L.) epidermis was bombarded with gold particles coated with plasmids using a Bio-Rad (Hercules) PDS-1000/He particle delivery system. Bombarded specimens were incubated for 24h on 0.5× Murashige and Skoog (MS) agar medium and observed using a LSM 5 Exciter confocal laser-scanning microscope (Carl Zeiss, Germany) with excitation at 488nm and emission at 505–530nm.

*Agrobacterium*-mediated transient expression of smGFP-tagged constructs in *N. benthamiana* leaves was used. *CaChitIV:GFP* or *CaChitIVΔSP:GFP* constructs under control of the CaMV 35S promoter were introduced into *A. tumefaciens* strain GV3101 by electroporation. Three days after infiltration with *Agrobacterium*, epidermal cells of *N. benthamiana* leaves were observed using a confocal laser-scanning microscope, as described above. The presence of GFP-tagged proteins was confirmed by immunoblotting using anti-GFP antibody.

### Immunoblotting

For Co-IP, total proteins were extracted from leaves using immunoprecipitation buffer [50mM HEPES (pH 7.5), 50mM NaCl, 10mM EDTA, 0.2% Triton X-100, and protease inhibitor cocktail (Roche, Mannheim, Germany)]; insoluble debris was pelleted by centrifuging leaf extracts at 15 000 *g* for 30min at 4 °C. The soluble protein extracts were incubated with monoclonal anti-cMyc or anti-HA agarose conjugates (Sigma-Aldrich, St Louis, MO, USA) overnight. Beads were collected and washed three times with wash buffer [50mM HEPES (pH 7.5), 50mM NaCl, 10mM EDTA, 0.1% Triton X-100, and protease inhibitor cocktail (Roche)]. Eluted proteins were analysed using immunoblotting with anti-cMyc or anti-HA peroxidase conjugates. Immunodetection was performed using the WEST-ZOL plus protein gel blot detection system, according to the manufacturer’s instructions (INTRON, Seoul, Korea).

### RNA gel blot and quantitative reverse transcription–PCR (RT–PCR) analyses

Total RNA was extracted from pepper plants using Isol-RNA lysis reagent (5 Prime, Gaithersburg, MD, USA), according to the manufacturer’s instructions. Total RNA (20 μg) was denatured by heating at 65 °C for 10min in a formaldehyde gel loading buffer and then separated by electrophoresis on 7.4% formaldehyde/1.2% agarose gels. Gels were immersed in deionized water for 30min and RNA transferred to Hybond™-N^+^ membranes (Amersham, Little Chalfont, UK), followed by cross-linking under UV illumination.

To generate the *CaChitIV* gene-specific probe, full-length *CaChitIV* cDNA was labelled with [^32^P[dCTP using the Klenow fragment of DNA polymerase 1 (Roche). Membranes were pre-hybridized and then hybridized overnight with the probe at 65 °C. After hybridization, the membranes were washed twice with 2× SSC, 0.1% SDS for 10min at room temperature and once with 0.1× SSC, 0.1% SDS for 15min. The membranes were exposed to X-ray film (Agfa, Mortsel, Belgium).

cDNA for real-time RT–PCR analysis was prepared using 1 μg of total RNA, 500ng of oligo dT(15) primer, and Moloney murine leukaemia virus reverse transcriptase at 42°C for 1h. Real-time PCR was performed with 1 μl of cDNA as a template and 45 reaction cycles, using iQTM SYBR Green Supermix and an iCycleriQ Real-Time PCR Detection System (Bio-Rad, Hercules, CA, USA), according to the manufacturer’s instructions. To normalize the transcript levels, *C. annuum* 18S rRNA and *CaACTIN* expression was monitored as reference genes in each reaction. The gene-specific primers used for the quantitative real-time RT–PCR analysis are listed in Supplementary Table S1 available at *JXB* online.

### *Agrobacterium*-mediated transient expression in pepper plants

The cMyc-tagged *CaPIK1* and HA-tagged *CaChitIV* were cloned into the pBIN35S plant binary vector, and *A. tumefaciens* strain GV3101 was transformed with the resulting plasmids using electroporation. The overnight cultures were centrifuged, harvested cells were diluted to OD_600_=1.0 in infiltration buffer (10mM MES, 10mM MgCl_2_, pH 5.7), and acetosyringone was added to a final concentration of 200 μM. The bacterial suspensions were infiltrated into the leaves of pepper plants at the six-leaf stage using a needleless syringe.

### Virus-induced gene silencing (VIGS)

*Tobacco rattle virus* (TRV)-based VIGS of *CaChitIV* and *CaPIK1* was performed in pepper plants (Liu *et al.*, 2002; Chung *et al.*, 2004). To elevate silencing specificity, 188bp and 195bp of non-conserved 5′ untranslated regions (UTRs) of the genes were PCR amplified and cloned into pCR2.1 TOPO vector (Invitrogen, Carlsbad, CA, USA), followed by their digestion with *Eco*RI. The eluates were inserted into the *Eco*RI site of pTRV2 to generate TRV2:*CaChitIV* and TRV2:*CaPIK1* constructs, respectively. Two weeks after sowing, expanded cotyledons of pepper plants were co-infiltrated with *A. tumefaciens* strain GV3101 transformed with VIGS vectors pTRV1 and pTRV2 containing *CaChitIV-UTR* and *CaPIK1-UTR*.

### *Arabidopsis* transformation

To induce constitutive *CaChitIV* overexpression (OX) in *Arabidopsis*, transgenic plants were generated using the floral dipping method (Clough and Bent, 1998). The *CaChitIV* coding region was amplified and inserted into *Xba*I/*Bam*HI sites of the binary vector pBIN35S under the control of the CaMV 35S promoter (Choi *et al.*, 2012). A pBIN35S:*CaChitIV* construct was introduced into *A. tumefaciens* strain GV3101 through electroporation. Transformants were selected on 0.5× MS agar plates containing 50 μg ml^–1^ kanamycin. Three transgenic *Arabidopsis* lines (#1, #2, and #3) were confirmed using RT–PCR analysis.

### Measurement of ion conductivity, H_2_O_2_, and NO bursts

Cell death was quantified by ion conductivity measurement. At various time points following bacterial infiltration, eight leaf discs (1.4cm in diameter) were excised and washed for 30min in 20ml of distilled water. After incubation for 3h in 20ml of distilled water, ion conductivity was measured using a Sension7 conductivity meter (Hach, Loveland, CO, USA).

H_2_O_2_ production in pepper leaves was quantified using the xylenol orange assay (Choi *et al.*, 2007). Briefly, the xylenol orange assay reagent was freshly prepared: 200 μl of solution [25mM FeSO_4_, 25mM (NH_4_)SO_4_ in 2.5M H_2_SO_4_] was added to 20ml of 125 μM xylenol orange in 100mM sorbitol. Eight leaf discs (0.5cm^2^) were floated on 1ml of distilled water in a microtube for 1h. After centrifugation for 1min at 12 000 *g*, 100 μl of supernatant was added to 1ml of xylenol orange assay reagent. The mixture was incubated for 30min at room temperature. H_2_O_2_ production was monitored by measuring the absorbance at 560nm using a DU 650 spectrophotometer (Beckman, Urbana, IL, USA). A standard curve was generated from measurements obtained from serial dilutions of H_2_O_2_ from 100 nmol to 100 μmol.

NO production was monitored using the NO-sensitive dye 4,5-diaminofluorescein diacetate (DAF-2DA; Sigma). Leaves were infiltrated with 200mM sodium phosphate buffer (pH 7.4) including 12.5 μM DAF-2DA, using a needleless syringe, and were incubated for 1h in the dark at room temperature. Fluorescence from diaminotriazolofluorescein (DAF-2T), the reaction product of DAF-2DA with NO, was observed using an LSM 5 Exciter confocal laser-scanning microscope (Carl Zeiss, Thornwood, NY, USA) with an excitation wavelength of 470nm; the emission images at 525nm were obtained at constant acquisition time. The fluorescence intensity of the digital image was determined by colour histogram analysis using Photoshop version 7.0 (Adobe, San Jose, CA, USA).

## Results

### CaPIK1 interacts with CaChitIV in yeast and *in planta*

The pepper receptor-like cytoplasmic protein kinase gene (*CaPIK1*) was previously isolated from pepper leaves infected with *Xcv* (Kim and Hwang, 2011). To identify proteins interacting with CaPIK1, *CaPIK1* was used as bait to screen a pepper cDNA library generated from avirulent *Xcv*-infected leaves using a GAL4-based yeast two-hybrid screen. Among the clones identified by screening, a pepper class IV chitinase, CaChitIV (accession no. KJ649334), was selected for further characterization as an interacting partner of CaPIK1 (Supplementary Fig. S1 at *JXB* online).

**Fig. 1.**
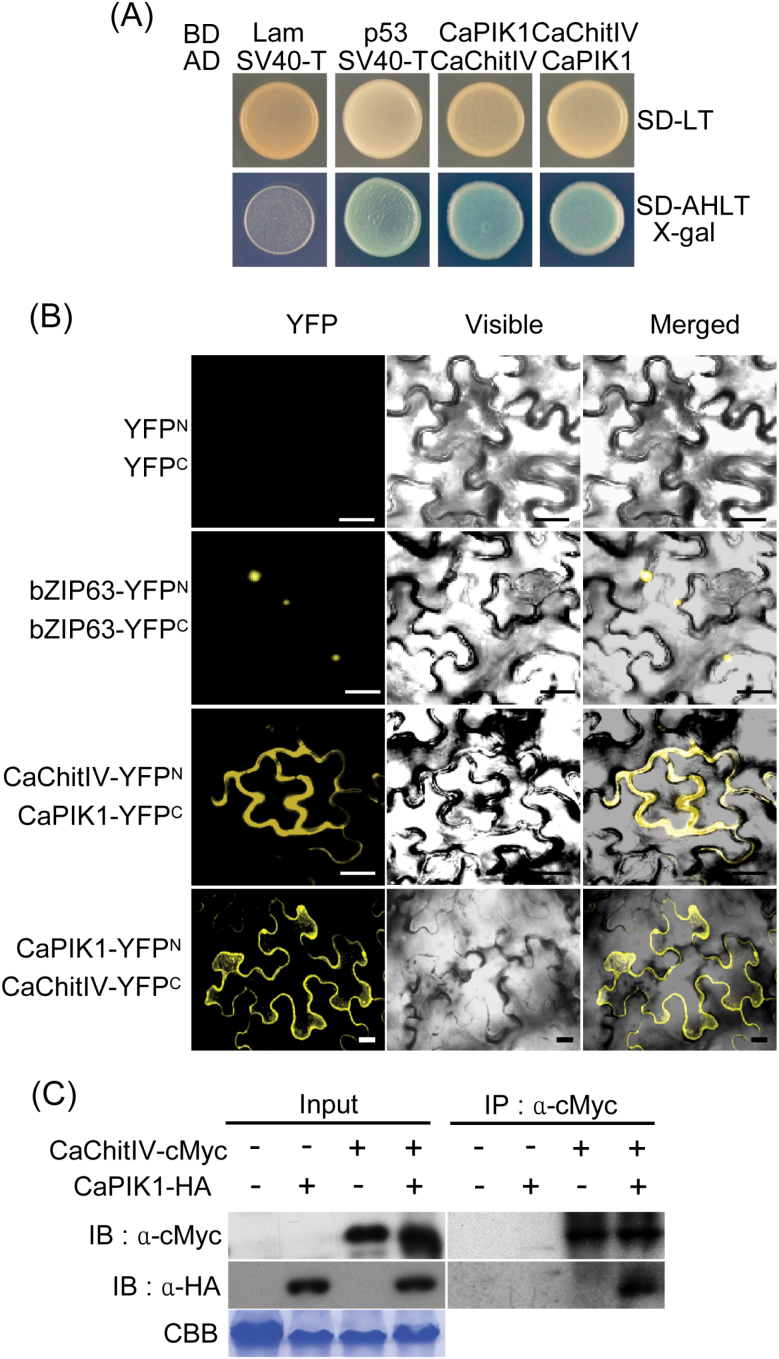
CaPIK1 interacts with CaChitIV in yeast and *in planta.* (A) Interaction of CaPIK1 with CaChitIV in a GAL4-based yeast two-hybrid system. Negative control: combination of human lamin C (BD/Lam) and SV40 large T antigen (AD/SV40-T) fusion constructs. Positive control: combination of murine p53 (BD/p53) and SV40 large T antigen (AD/SV40-T) fusion constructs. BD, GAL4 DNA-binding domain; AD, GAL4 activation domain; SD-LT, synthetic dropout medium lacking leucine (L) and tryptophan (T); SD-AHLT X-α-gal, synthetic dropout medium lacking adenine (A), histidine (H), leucine (L), and tryptophan (T) supplemented with X-α-Gal to monitor reporter gene expression. (B) Bimolecular fluorescent complementation (BiFC) analysis of the CaPIK1–CaChitIV interaction in *Nicotiana benthamiana*. Confocal images were taken from leaf epidermal cells; *bZIP63*-YFP^N^ and *bZIP63*-YFP^C^ constructs were used as positive controls. Scale bars=50 μm. (C) Co-immunoprecipitation (Co-IP) and immunoblot (IB) analyses of CaChitIV-cMyc and CaPIK1-HA co-expressed in *N. benthamiana* leaves. Protein loading is shown by Coomassie brilliant blue (CBB) staining. α-cMyc, cMyc antibody; α-HA, HA antibody. (This figure is available in colour at *JXB* online.)

The specific interaction between CaPIK1 and CaChitIV was verified by a vector change and re-transformation protocol. After recovering initial fusion constructs (BD/CaPIK1 and AD/CaChitIV) from positive colonies, pepper *CaPIK1* and *CaChitIV* cDNAs were re-introduced into pGADT7 and pGBKT7, respectively, to produce AD/CaPIK1 and BD/CaChitIV. The murine p53 (BD/p53), human lamin C (BD/Lam), and SV40 large T antigen (AD/T) were used as interaction controls. All the yeast constructs containing the indicated combinations of plasmids grew on synthetic dropout (SD) medium lacking leucine (L) and tryptophan (T). The yeast combination BD/CaPIK1 and AD/CaChitIV grew well on plates lacking adenine, histidine, leucine, and tryptophan (SD-AHLT), as did the combination BD/CaChitIV and AD/CaPIK1; both combinations showed blue colour on the X-α-Gal plate, as did a positive control harbouring BD/p53 and AD/SV40-T (Fig. 1A). This indicates that CaPIK1 specifically interacts with CaChitIV in the GAL4-based yeast two-hybrid system.

The interaction of CaPIK1 and CaChitIV *in planta* was examined using BiFC analysis (Walter *et al.*, 2004). BiFC vectors (pSPYNE and pSPYCE) containing YFP^N^ and YFP^C^, respectively, were used to construct CaPIK1–YFP^N^ and CaChitIV–YFP^C^, or vice versa. Interactions between the fusion proteins were visualized in *N. benthamiana* leaves using *Agrobacterium*-mediated transient co-expression. Combinations of CaChitIV–YFP^N^ and CaPIK1–YFP^C^, or vice versa, were observed in the cytoplasm and the plasma membrane, indicating that CaPIK1 binds to CaChitIV in plant cells (Fig. 1B). A combination of bZIP63–YFP^N^ and bZIP63–YFP^C^ was used as a nuclear-localized BiFC control (Walter *et al.*, 2004; Fig. 1B).

The CaPIK1 and CaChitIV interaction *in planta* was further confirmed using Co-IP (Fig. 1C). cMyc-tagged *CaChitIV* and/or HA-tagged *CaPIK1* were transiently expressed in *N. benthamiana* leaves. Three days after infiltration, proteins were extracted from leaves and incubated with monoclonal anti-cMyc agarose conjugates to immunoprecipitate CaChitIV. After immunoprecipitation, potential CaChitIV and CaPIK1 complexes were separated using SDS–PAGE. CaPIK1-HA was detected only when co-expressed with CaChitIV-cMyc. These results indicate that CaPIK1 physically interacts with CaChitIV in plant cells.

### Sequence and expression analysis of *CaChitIV* in pepper

*CaChitIV* (accession no. KJ649334) is a 990bp cDNA encoding a chitinase of 277 amino acids (Supplementary Fig. S1 at *JXB* online). A BLAST search found that the CaChitIV protein sequence closely resembled other plant chitinases (Supplementary Fig. S2A), being 82% identical to *Nicotiana tabacum* chitinase (accession no. BAF44533), 61% identical to *Oryza sativa* chitinase (accession no. NP_001053186), 57% identical to *Zea mays* chitinase (accession no. NP_001158904; Chaudet et al., 2004), and 53% identical to *Picea abies* chitinase (accession no. AY270017; Ubhayasekera *et al.*, 2009). As shown in Supplementary Fig. S2B, CaChitIV contains a signal peptide with the initiation methionine (amino acids 1–28), a CBD (amino acids 30–61), and a glycol hydrolase domain (glyco-hydro-19; amino acids 77–277), indicating that *CaChitIV* encodes an extracellular chitinase.

It was previously shown that *CaPIK1* is constitutively expressed in flowers but either not at all or weakly in leaves, fruits, stems, and roots of healthy pepper plants (Kim and Hwang, 2011). However, *CaPIK1* expression is strongly induced in pepper leaves by infection with virulent (Ds1) and avirulent (Bv5-4a) strains of *Xcv* (Kim and Hwang, 2011). In the present study, RNA gel blot analysis was used to investigate transcriptional regulation of *CaChitIV*, a *CaPIK1*-interacting partner, in pepper plants.

**Fig. 2.**
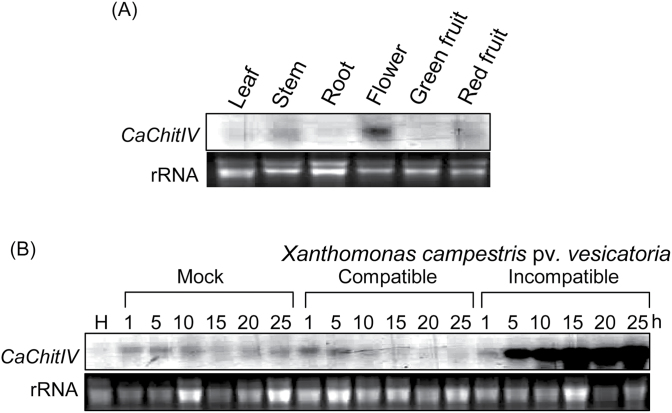
RNA gel blot analysis of expression of *CaChitIV* in pepper plants at the six-leaf stage. (A) Constitutive expression of *CaChitIV* in various organs of pepper plants. (B) Time-courses of induction of *CaChitIV* in leaf tissues following inoculation with virulent (compatible) Ds1 or avirulent (incompatible) Bv5-4a strains of *Xanthomonas campestris* pv. *vesicatoria*. The blot was hybridized with a ^32^P-labelled probe against the full-length *CaChitIV* cDNA. Equal loading (20 μg per lane) of RNA was verified by staining with ethidium bromide. H, healthy leaves.

*CaChitIV* was constitutively expressed in flowers but detected only at relatively low levels in leaves, stems, green fruits, and red fruits (Fig. 2A). It was next investigated whether *CaChitIV* transcription is altered by *Xcv* infection (Fig. 2B). Infection with avirulent (incompatible) Bv5-4a *Xcv* rapidly and strongly induced expression of *CaChitIV* in pepper leaves. In contrast, weak induction of *CaChitIV* expression was seen in mock-inoculated leaves and in leaves infected with virulent (compatible) *Xcv* Ds1.

### CaChitIV localizes to the endoplasmic reticulum

The first 18 amino acids of CaChitIV form an N-terminal signal peptide (Supplementary Fig. S2B at *JXB* online). Such signal peptides cause proteins to be targeted to the secretory pathway through organelles including the ER, Golgi body, or endosomes (Blobel and Dobberstein, 1975; Crowley *et al.*, 1994; Johnson *et al.*, 2013). To determine the subcellular localization of CaChitIV, C-terminal smGFP-tagged *CaChitIV* and signal peptide-deleted *CaChitIV* (*CaChitIVΔSP*) were constructed. Using *Agrobacterium*-mediated transient expression, *CaChitIV* and *CaChitIVΔSP* fusion proteins with GFP were expressed in *N. benthamiana*.

**Fig. 3.**
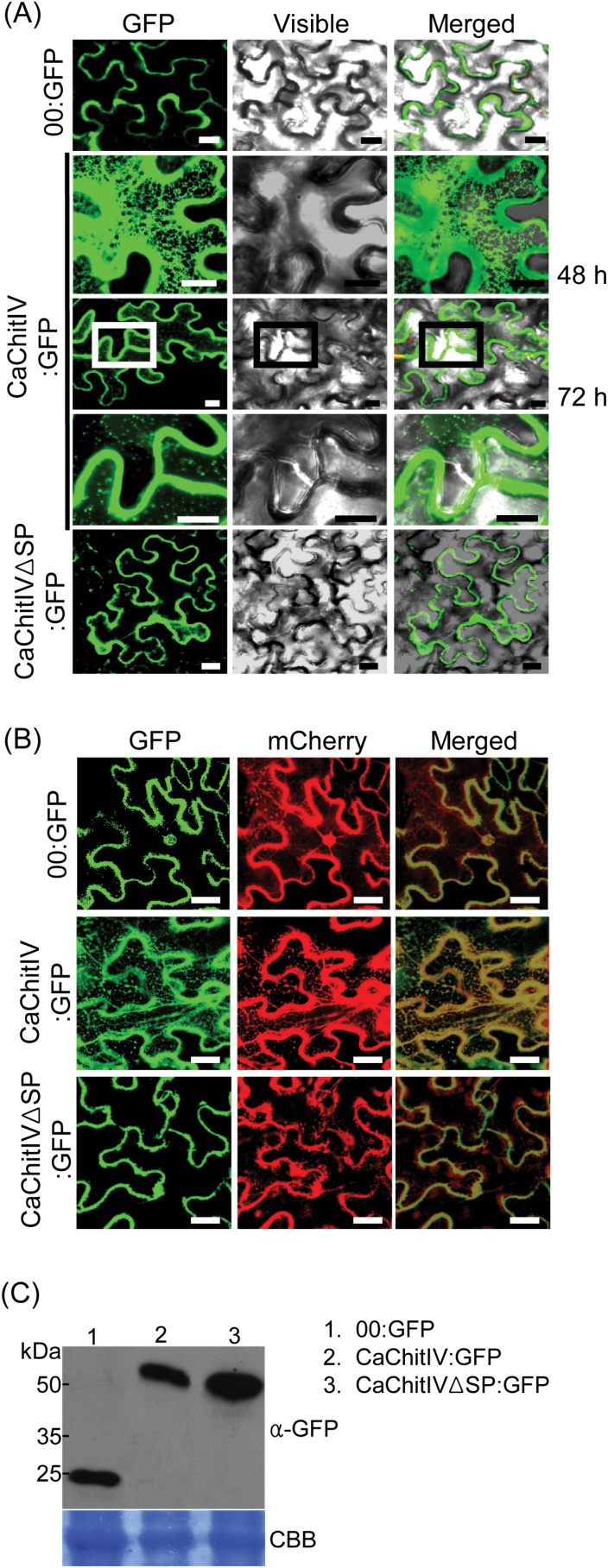
Subcellular localization of 35S:00:GFP, 35S:CaChitIV:GFP, and 35S:CaChitIVΔSP:GFP transiently expressed in *Nicotiana benthamiana* leaves. (A) Detection of transient expression by confocal laser-scanning microscopy of leaf epidermal cells containing *GFP*-tagged constructs 3 d after infiltration with *Agrobacterium*. Scale bars=20 μm. (B) Detection of CaChitIV in the endoplasmic reticulum (ER). mCherry indicates the localization of the ER marker, ER-rk*CD3-959*. Scale bars=20 μm. (C) Immunoblot analysis of GFP, CaChitIV:GFP, and CaChitIVΔSP proteins in leaf extracts. Total leaf proteins were subjected to immunoblotting using anti-GFP (α-GFP) antibody. Equal protein loading is indicated by Coomassie brilliant blue (CBB) staining. (This figure is available in colour at *JXB* online.)

At 48h after infiltration with *Agrobacterium*, CaChitIV:GFP expression was observed exclusively in polygonal net-like structures (Fig. 3A); however, by 72h after infiltration, GFP signals were strongly detected in the cell periphery and apoplastic regions. Transient expression of CaChitIVΔSP:GFP in epidermal cells of *N. benthamiana* leaves was similar to that of the non-fused GFP (00:GFP) control, which was dispersed throughout the cytosol and nucleus (Fig. 3A). In onion epidermal cells, CaChitIV:GFP was localized as a membrane-bound spot at the cell periphery 24h after bombardment (Supplementary Fig. S3A, B at *JXB* online); whereas, after 48h, GFP signals were detected exclusively at the cell periphery and apoplastic regions.

The subcellular distribution of CaChitIV:GFP in net-like structures (Fig. 3A) resembles that of plant ER marker proteins, such as *Arabidopsis* Ca^2+^-ATPase, isoform 2 protein (ACA2p), and *A. thaliana* wall-associated kinase 2 (AtWAK2) (He *et al.*, 1999; Bracha *et al.*, 2002). To investigate whether CaChitIV:GFP also localizes to the ER, the ER marker, ER-rk*CD3-959*, which was created by adding the AtWAK2 signal peptide to the N-terminus of the mCherry fluorescent protein and the ER retention signal, His-Asp-Glu-Ler, to its C-terminus (Nelson *et al.*, 2007), was used. *00:GFP,CaChitIV:GFP* or *CaChitIVΔSP:GFP* were transiently co-expressed with *ER-rk CD3-959* in leaf epidermal cells of *N. benthamiana* (Fig. 3B). Co-localization of the fusion proteins, CaChitIV:GFP and ER-rk CD3-959, was observed indicating that CaChitIV:GFP localizes to the ER. In contrast, free-GFP and CaChitIVΔSP:GFP were detected only in the cytoplasm and nucleus of the same leaves (Fig. 3B). Transient expression of *00:GFP*, *CaChitIV:GFP*, and *CaChitIVΔSP:GFP* in *N. benthamiana* leaves was confirmed by immunoblotting with GFP antibodies (α-GFP; Fig. 3C). Collectively, these results indicate that the signal peptide CaChitIV targets the protein to the ER.

### Co-expression of *CaChitIV* with *CaPIK1* promotes *CaPIK1*-triggered cell death and defence responses

**Fig. 4.**
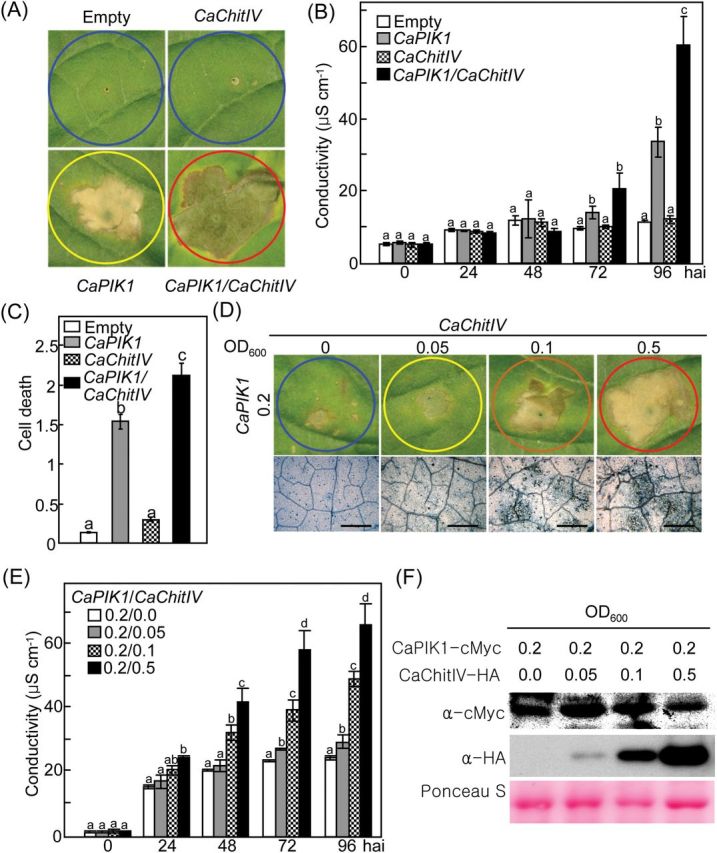
Transient co-expression of *CaChitIV* with *CaPIK1* in pepper leaves enhances the *CaPIK1*-triggered cell death response. (A) Cell death phenotypes, 3 d after infiltration with *Agrobacterium*, of pepper leaves transiently expressing empty vector control, *CaPIK1*, *CaChitIV*, or *CaPIK1/CaChitIV*. (B) Electrolyte leakage from leaf discs at different time points after infiltration. (C) Quantification of cell death in pepper leaves transiently expressing empty vector control, *CaPIK1*, *CaChitIV*, or *CaPIK1/CaChitIV* 3 d after agro-infiltration (OD_600_=1.0). (D) Cell death phenotypes and trypan blue-stained cells in leaves transiently expressing *CaPIK1* and *CaChitIV* 3 d after infiltration with different inoculum concentrations of *Agrobacterium*. Cell death levels were rated based on a 0–3 scale: 0, no cell death (<10%); 1, weak cell death (10–30%); 2, partial cell death (30–80%); and 3, full cell death (80– 100%). (E) Electrolyte leakage from leaf discs at different time points following infiltration with different inoculum concentrations. (F) Immunodetection of CaPIK1-cMyc and CaChitIV-HA expression following infiltration with different inoculum concentrations; immunoblotting was performed with cMyc antibody (α-cMyc) and HA antibody (α-HA), respectively. Equal protein loading is shown by Ponceau S staining. For B, C, and E, data are the means ±standard deviations from three independent experiments. Statistically significant differences in B, C, and E, according to Fisher’s least significant difference (LSD) test (*P*<0.05), are indicated by the letters above the data points. hai, hours after infiltration. (This figure is available in colour at *JXB* online.)

Transient expression of *CaPIK1* in pepper leaves triggers early defencse responses, including ROS and NO bursts, and ultimately leads to HR-like cell death (Kim and Hwang, 2011). In the present study, it was investigated whether co-expression of *CaChitIV* with *CaPIK1* in pepper leaves influenced the *CaPIK1*-triggered cell death response (Fig. 4).

Transient expression of empty vector or *CaChitIV* did not trigger the cell death response (Fig. 4A). Electrolyte leakage from pepper leaves co-expressing *CaPIK1* and *CaChitIV* was significantly greater than that from leaves expressing *CaPIK1*alone (Fig. 4B), indicating an enhanced level of necrosis. Moreover, co-expression of *CaChitIV* with *CaPIK1* effectively enhanced *CaPIK1*-triggered HR-like cell death responses (Fig. 4A, C), indicating that CaChitIV positively regulates cell death induction by CaPIK1. Cell death responses were classified based on a 0–3 scale: 0, no cell death (<10%); 1, weak cell death (10–30%); 2, partial cell death (30–80%); and 3, full cell death (80–100%) (Choi and Hwang, 2011). The effect of co-expression of *CaChitIV* with *CaPIK1* on the level of cell death was higher than that of *CaPIK1* expression alone (Fig. 4C). The synergistic effects of *CaChitIV* on *CaPIK1*-mediated cell death in pepper were further investigated in leaves by co-expressing *CaPIK1* at a low inoculum density (OD_600_=0.2) with different inoculum concentrations (OD_600_=0.05, 0.1, and 0.5) of *CaChitIV*. Increasing the inoculum density of *CaChitIV* enhanced cell death levels, trypan blue-stained cell death response, and electrolyte leakage (Fig. 4D, E). Expression of CaPIK1 and proteins was confirmed by immunoblot analysis (Fig. 4F). Increased inoculum concentration of *CaChitIV* gradually increased the level of CaChitIV expression in pepper leaves.

**Fig. 5.**
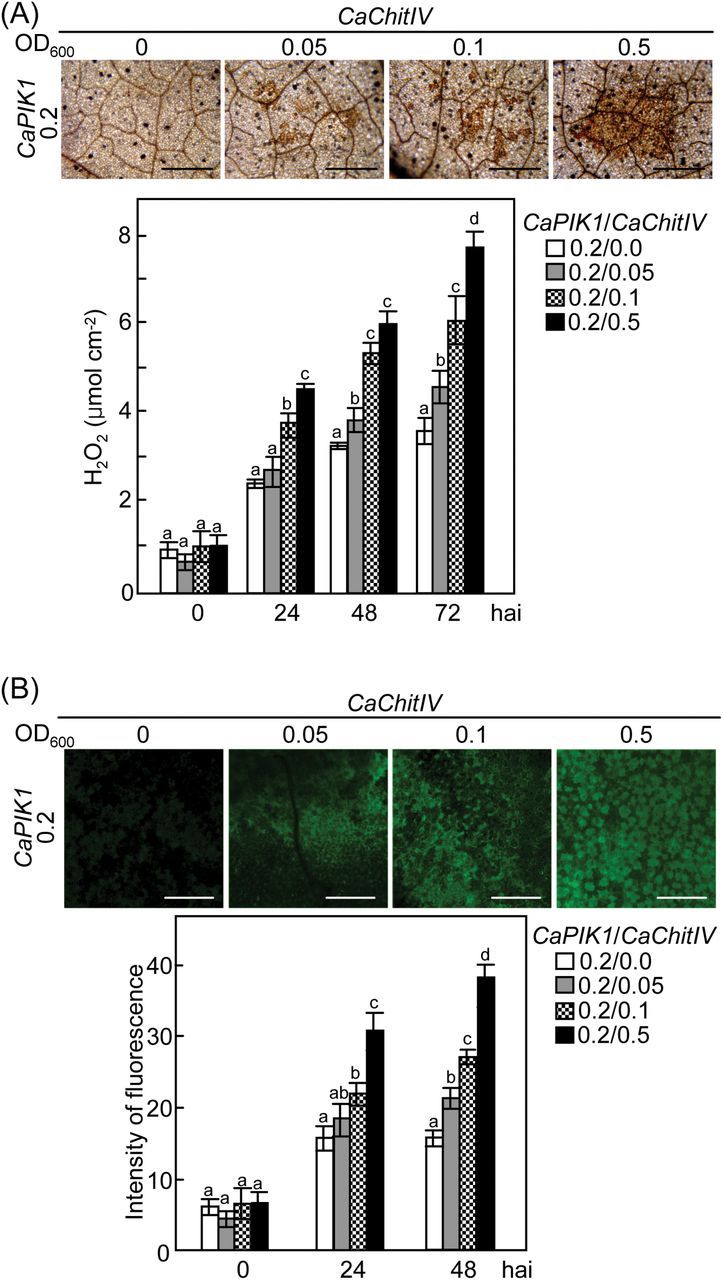
Transient co-expression of *CaPIK1* and *CaChitIV* in pepper leaves enhances bursts of ROS and NO. (A) 3,3′-Diaminobenzidine (DAB) staining and quantification of H_2_O_2_ accumulation in leaves at the stated time points after infiltration with *Agrobacterium*. Data are the means ±standard deviations from three, independent experiments. (B) Quantification of NO production in leaves after infiltration with *Agrobacterium*. The infiltrated leaf areas were visualized 1h after infiltration using a confocal microscope. Fluorescence intensities were quantified by colour histogram analysis. Data are means ±standard deviations from 30 randomly taken pictures. Scale bars=500 μm. Letters above the bars in A and B indicate significant differences between treatments, as determined by the LSD test (*P*<0.05). hai, hours after infiltration. (This figure is available in colour at *JXB* online.)

In addition, the effect of co-expressing *CaChitIV* with *CaPIK1* on ROS (Fig. 5A) and NO bursts (Fig. 5B) in pepper leaves was investigated. ROS are known to act as regulators of PCD in animal and plant cells (Jabs, 1999; Jones, 2001; Doyle *et al.*, 2010) and NO is a reactive nitrogen species acting as an intermediate in multiple signalling pathways in plants (Besson-Bard *et al.*, 2008). Levels of H_2_O_2_ and NO gradually increased in pepper leaves transiently co-expressing *CaPIK1* and *CaChitIV*, in proportion to the original inoculum density of *Agrobacterium*.

**Fig. 6.**
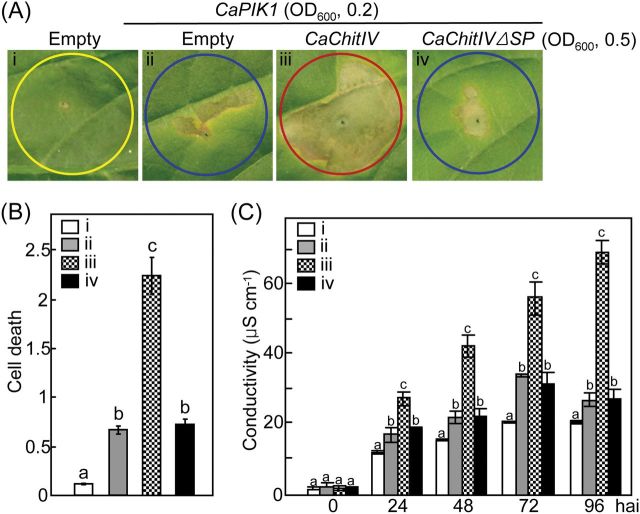
Transient co-expression of *CaChitIVΔSP* with *CaPIK1* does not enhance the *CaPIK1*-triggered cell death response in pepper leaves. (A) Cell death phenotypes in pepper leaves transiently expressing empty vector control, *CaChitIV*, or *CaChitIVΔSP* in combination with *CaPIK1* 2 d after infiltration with *Agrobacterium*. Cell death levels were rated based on a 0–3 scale: 0, no cell death (<10%); 1, weak cell death (10–30%); 2, partial cell death (30–80%); and 3, full cell death (80–100%). (B) Quantification of cell death in pepper leaves treated as in A. (C) Electrolyte leakages from leaf discs at the stated time points following infiltration. Data in B and C are the means ±standard deviations from three, independent experiments. Statistically significant differences between treatments, according to the LSD test (*P*<0.05), are indicated by the letters above the bars. i, Empty vector; ii, *CaPIK1*/Empty; iii, *CaPIK1/CaChitIV*; iv, *CaPIK1*/*CaChitIVΔSP*. hai, hours after infiltration. (This figure is available in colour at *JXB* online.)

*CaChitIVΔSP*:GFP expression was restricted to the cytoplasm and nuclei of leaf epidermal cells of *N. benthamiana* (Fig. 3B). It was therefore investigated whether co-expression in pepper leaves of *CaChitIVΔSP* with *CaPIK1* affected the *CaPIK1*-triggered cell death response (Fig. 6). In contrast to co-expression with *CaChitIV*, co-expression of *CaPIK1* with *CaChitIVΔSP* did not enhance the *CaPIK1*-triggered HR-like cell death response (Fig. 6A, B). Electrolyte leakage from pepper leaves co-expressing empty vector control or *CaChitIVΔSP* in combination with *CaPIK1* was significantly lower than that from leaves co-expressing *CaChitIV* and *CaPIK1* (Fig. 6C). Thus expression of *CaChitIVΔSP* in the cytoplasm and nucleus does not increase levels of *CaPIK1*-triggered cell death.

### Silencing *CaChitIV* and/or *CaPIK1* in pepper plants increases susceptibility to *Xanthomonas campestris* pv. *vesicatoria* infection

**Fig. 7.**
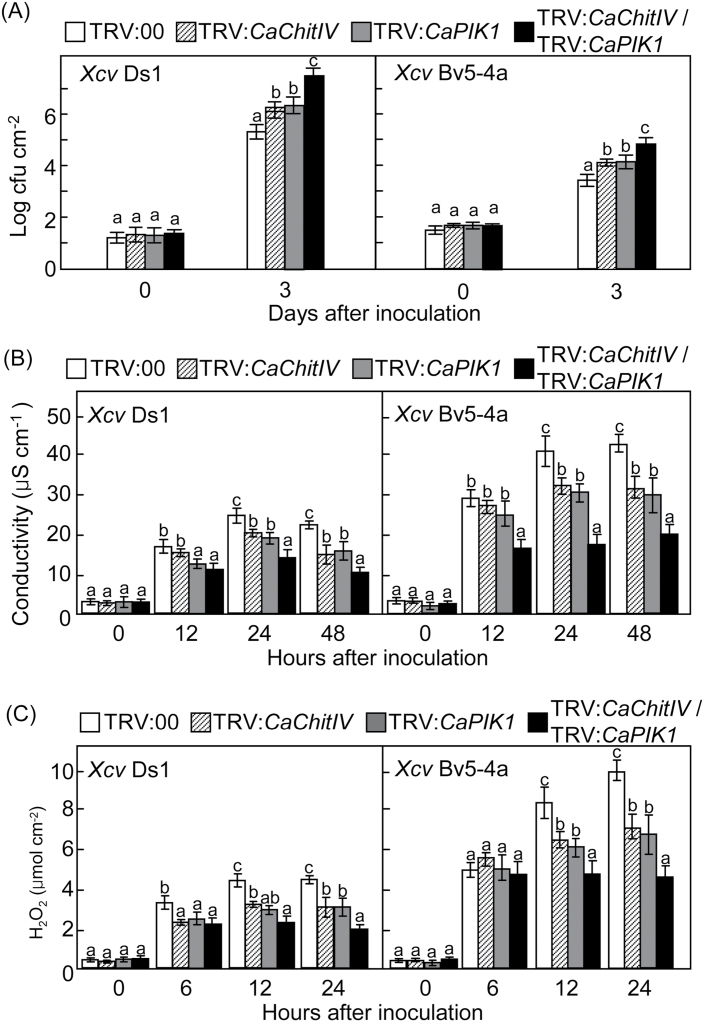
Silencing of *CaChitIV* and/or Ca*PIK1* in pepper plants enhances susceptibility to infection with virulent Ds1 (compatible) and avirulent Bv5-4a (incompatible) strains of *Xanthomonas campestris* pv. *vesicatoria* (*Xcv*). (A) Bacterial growth in leaves of pepper plants infiltrated with empty vector control (TRV:*00*) or with gene silencing constructs TRV:*CaChitIV*, TRV:*CaPIK1*, or TRV:*CaChitIV*/TRV:*CaPIK1*, and then infected with *Xcv* (5×10^4^ cfu ml^–1^). (B) Quantification of electrolyte leakage from leaves infected with *Xcv* (5×10^7^ cfu ml^–1^). (C) Quantification of H_2_O_2_ accumulation in leaves infected with *Xcv* (5×10^7^ cfuml^–1^). Data are means ±standard deviations from three, independent experiments. Letters above the bars in A–C indicate statistically significant differences between treatments, according to the LSD test (*P*<0.05).

To investigate the effect of loss of *CaChitIV* and/or *CaPIK1* function, VIGS (Liu *et al*., 2006) was used to generate pepper plants in which expression of *CaChitIV*, *CaPIK1*, or both *CaChitIV* and *CaPIK1* had been silenced. Expression of *CaChitIV* and/or *CaPIK1* was significantly down-regulated during *Xcv* infection in pepper leaves in which *CaChitIV* and/or *CaPIK1* was silenced, indicating that *CaChitIV* and/or *CaPIK1* were efficiently silenced (Fig. 9). It was observed that growth of virulent and avirulent *Xcv* reached significantly higher levels in leaves from gene-silenced plants than in leaves from empty vector control plants (Fig. 7A). Notably, silencing both *CaChitIV* and *CaPIK1* allowed the proliferation of virulent Ds1 and avirulent Bv5-4a strains of *Xcv* over and above the effect of silencing each gene separately. This indicates that *CaChitIV* expression contributes to the *CaPIK1-*mediated basal defence and HR-like cell death response.

**Fig. 8.**
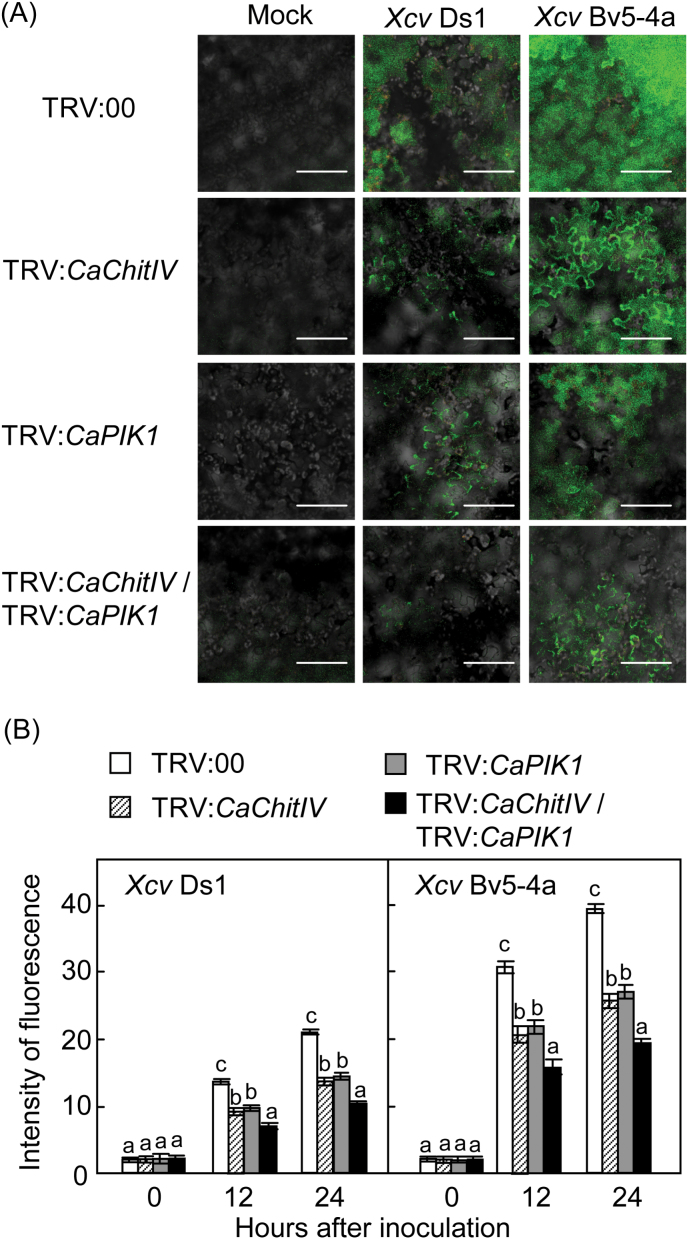
Microscopic images and quantification of NO production in empty vector control and *CaChitIV* and/or Ca*PIK1-*silenced pepper leaves infected with virulent (compatible) Ds1 and avirulent (incompatible) Bv5-4a strains of *Xanthomonas campestris* pv. *vesicatoria* (*Xcv*) (5×10^7^ cfu ml^–1^). (A) Visualization of the leaf areas 18h after infiltration with *Xcv* using a confocal microscope. Mock: infiltrated with 10mM MgCl_2_. Scale bars=100 μm. (B) Quantification of fluorescence intensities in leaves by colour histogram analysis. Data are means ±standard deviations from 30 randomly taken pictures. Letters above the bars indicate statistically significant differences between treatments, according to the LSD test (*P*<0.05).

The cell death and defence phenotypes were substantiated by an electrolyte leakage assay (Fig. 7B). Avirulent *Xcv* infection resulted in a higher level of electrolyte leakage from leaf discs than the virulent *Xcv* infection; however, electrolyte leakage from leaves in which *CaChitIV*, *CaPIK1*, or *CaChitIV* and *CaPIK1* were silenced was significantly less than from leaves containing the empty vector control, following both virulent and avirulent *Xcv* infection. Notably, silencing of both *CaChitIV* and *CaPIK1* significantly reduced electrolyte leakage from leaf discs infected with *Xcv*. To determine whether the silencing of *CaChitIV* and/or *CaPIK1* inhibited ROS accumulation, H_2_O_2_ production in pepper leaves was quantified using the xylenol orange assay (Choi *et al.*, 2007). At the early stage of *Xcv* infection, significantly lower levels of H_2_O_2_ accumulated in leaves in which expression of both *CaChitIV* and *CaPIK1* was silenced than in leaves containing the empty vector control or leaves in which *CaChitIV* or *CaPIK1* had been silenced (Fig. 7C). To determine whether the silencing of *CaChitIV* and/or *CaPIK1* inhibited NO accumulation, NO production in pepper leaves was visualized using the NO-sensitive dye DAF-2DA (Fig. 8A). During both virulent and avirulent *Xcv* infection, significantly lower levels of NO accumulated in both *CaChitIV-* and *CaPIK1-*silenced leaves than in the empty vector control and *CaChitIV-* or *CaPIK1-*silenced leaves (Fig. 8B). Together, these results indicate that *CaChitIV* and *CaPIK1* co-expression triggers pathogen-induced hypersensitive cell death, and ROS and NO bursts in pepper leaves.

**Fig. 9.**
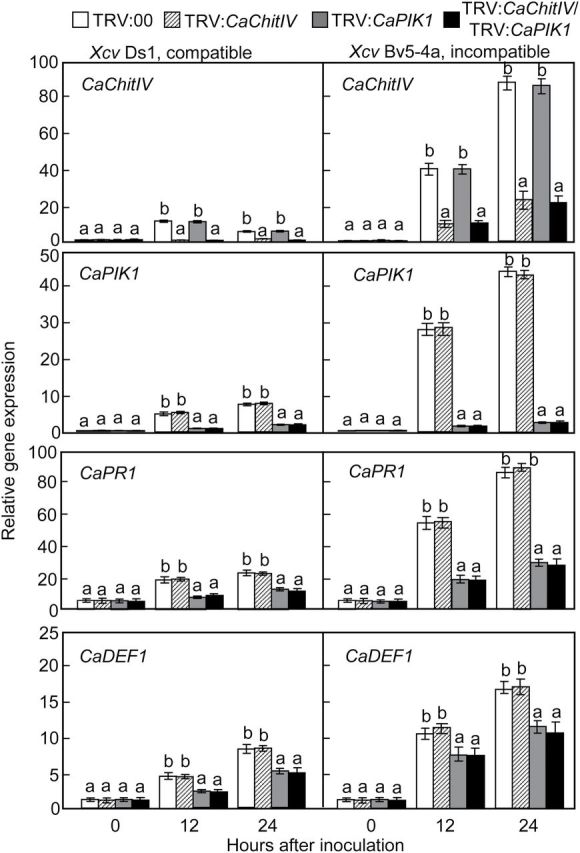
Quantitative real-time RT–PCR analysis of relative gene expression of *CaChitIV*, *CaPIK1*, *CaPR1*, and *CaDEF1* in pepper plants infected with virulent Ds1 (compatible) or avirulent Bv5-4a (incompatible) strains of *Xanthomonas campestris* pv. *vesicatoria* (*Xcv*). *CaPR1*, pathogenesis-related protein; *CaDEF1*, defensin. Expression values were normalized by the expression levels of *Capsicum annuum CaACTIN*. Data are the means ±standard deviations from three, independent experiments. Letters above the bars indicate statistically significant differences between treatments, according to the LSD test (*P*<0.05). (This figure is available in colour at *JXB* online.)

To investigate whether silencing of *CaChitIV* and/or *CaPIK1* affects the expression of defence-related genes in pepper, quantitative real-time RT–PCR analysis was performed (Fig. 9; Supplementary Fig. S4 at *JXB* online). Expression values of these genes were normalized by the expression levels of *C. annuum CaACTIN* and *18S rRNA* as reference genes. *CaPIK1* silencing significantly compromised the induction of *CaPR1* (PR1) and *CaDEF1* (defensin), but not *CaChitIV* (chitinase IV), during virulent and avirulent *Xcv* infection. In contrast, induction of these three defence response genes during *Xcv* infection was not reduced in *CaChitIV*-silenced plants (Fig. 9). However, silencing of both *CaChitIV* and *CaPIK1* significantly inhibited induction of these defence response genes following infection. These results indicate that although *CaPIK1* expression positively regulates expression of defence-related genes in pepper, *CaChitIV* may not play a significant role in the regulation of downstream defence gene expression.

### Overexpression of *CaChitIV* in *Arabidopsis* reduces susceptibility to *Hyaloperonospora arabidopsidis* infection

**Fig. 10.**
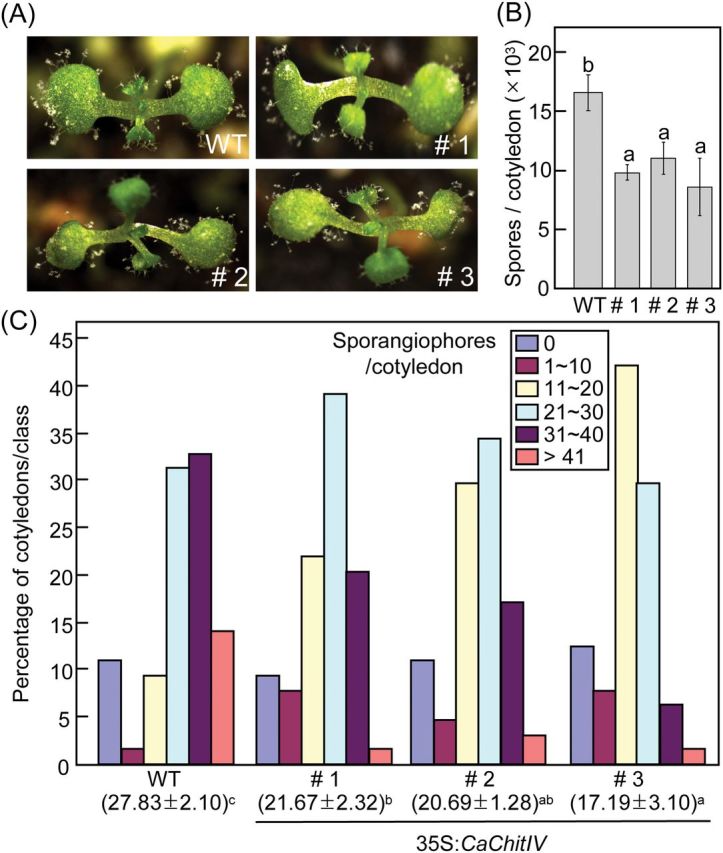
Overexpression of *CaChitIV* in *Arabidopsis* reduces susceptibility to infection with *Hyaloperonospora arabidopsidis* isolate Noco2. (A) Disease symptoms on cotyledons 7 d after infection with *H*. *arabidopsidis* (5×10^4^ conidiospores ml^–1^). (B) Mean number of spores on the cotyledons of wild-type (WT) and *CaChitIV-OX* plants. (C) Quantification of sporangiophores on at least 50 cotyledons of wild-type and *CaChitIV-OX* plants 7 d after inoculation. The mean number of sporangiophores ±standard deviation is shown below each of the lines tested. Values in B and C are means ±standard deviations from three independent experiments. Different letters indicate statistically significantly differences between groups, according to the LSD test (*P*<0.05).

To investigate whether increased expression of *CaChitIV* affected resistance to *Hpa* infection, wild-type (Col-0) and transgenic *Arabidopsis* plants overexpressing *CaChitIV* (*CaChitIV-OX*) were inoculated with *Hpa* isolate Noco2 (Fig. 10). RT–PCR analysis showed that *CaChitIV* was constitutively overexpressed in leaves of transgenic lines #1, #2, and #3 (Supplementary Fig. S5 at *JXB* online).

More vigorous growth of *Hpa* isolate Noco2 was observed on cotyledons of wild-type seedlings than on seedlings from *CaChitIV*-*OX* lines (Fig. 10A). Seven days after inoculation, *Hpa* had produced significantly fewer conidiospores when grown on the cotyledons of *CaChitIV*-*OX* seedlings than on wild-type cotyledons (Fig. 10B). *Hpa*-infected cotyledons were grouped into five classes based on the number of sporangiophores per cotyledon: 0, 1–10, 11–20, 21–30, 31–40, and >41. The incidence of classes containing lower numbers of sporangiophores was significantly higher for *CaChitIV*-*OX* lines than for wild-type plants (Fig. 10C). Collectively, these results indicate that *CaChitIV* overexpression confers increased basal resistance to *Hpa* infection in *Arabidopsis*.

## Discussion

In a previous work, it was reported that the receptor-like cytoplasmic protein kinase, CaPIK1, acts as a positive regulator to trigger an HR-like cell death response in pepper plants, as well as accumulation of ROS and NO (Kim and Hwang, 2011). Here, evidence is provided that the pepper class IV chitinase, CaChitIV, interacts with CaPIK1 in yeast and *in planta*. Using *Agrobacterium*-mediated transient co-expression of *CaChitIV* and *CaPIK1*, a critical role for *CaChitIV* in *CaPIK1*-triggered cell death and defence responses was revealed.

It is well known that plant chitinases function in plant defence responses to fungal and oomycete pathogen infection (Collinge *et al.*, 1993; Kim and Hwang, 1994; Lee *et al.*, 2000). Chitinase is a catalytic enzyme responsible for the hydrolysis of chitin, a linear polymer of GlcNAc and an important structural component of the fungal cell wall (Wubben *et al.*, 1992; Nielsen *et al.*, 1993). However, chitinase gene expression in plants is also induced by infection with viruses, bacteria, and oomycetes that do not contain chitin or related structures (Métraux *et al.*, 1988; Hong *et al.*, 2000; Hong and Hwang, 2002; Ott *et al.*, 2006). Here, strong induction of *CaChitIV* expression was shown at an early stage of infection with avirulent *Xcv* Bv5-4a carrying AvrBsT that induces cell death and defence responses in pepper (Kim *et al.*, 2010). However, infection with a virulent strain of *Xcv* does not induce expression of *CaChitIV*. Induced chitinases may be utilized as a positive regulator of early basal resistance. Non-pathogenic, saprophytic, and avirulent bacteria triggered early basal resistant responses, such as induction and accumulation of chitinases in tobacco plants, whereas virulent bacterial infection suppressed chitinase activity (Ott *et al.*, 2006), indicating that virulent bacteria have molecular mechanisms to circumvent early basal resistance and so ensure their survival in host tissues. However, little is known about how chitinases regulate basal resistance and HR-like cell death in response to infection with avirulent bacterial pathogens.

Co-expression of *CaChitIV* with *CaPIK1* accelerated the *CaPIK1*-triggered cell death response. The presence of a signal peptide led to the prediction that CaChitIV would be secreted to the extracellular, apoplastic region via the ER. A subcellular localization assay revealed that CaChitIV protein was indeed localized mainly in the ER; in contrast, *CaChitIVΔSP*:GFP, lacking the signal peptide, localized to the cytoplasm and nucleus. Co-expression with *CaChitIVΔSP* did not enhance the *CaPIK1*-triggered HR-like cell death response. These results suggest that it is localization of *CaChitIV* to the ER that is responsible for its enhancement of *CaPIK1*-triggered cell death.

Expression of CaPIK1 protein *in planta* maintained a steady-state level following inoculation with a given dose of *Agrobacterium* (OD_600_=0.2); however, CaChitIV levels increased with the cell density of the inoculum, indicating that CaPIK1 and CaChitIV do not affect each other at the protein level. When *CaPIK1* and *CaChitIV* were co-expressed in pepper leaves, induction of ROS and NO accumulation gradually increased, in line with increasing density of *CaChitIV*-containing inoculum. Such an enhancement of ROS and NO bursts following increased *CaChitIV* expression may lead to the promotion of *CaPIK1*-triggered cell death, as ROS are known to work synergistically with NO to stimulate PCD and assist in defence response to pathogens (Besson-Bard *et al.*, 2008; Perchepied *et al.*, 2010). The enhanced ROS and NO bursts seen in pepper leaves co-expressing *CaPIK1* and *CaChitIV* support the suggestion that ROS and NO act as signalling radicals in plant cell death and defence responses, including MAPK activation, expression of defence-related genes, and cell wall thickening via callose accumulation (Torres and Dangl, 2005; Asai *et al.*, 2008).

The TRV-based VIGS system (Liu *et al.*, 2002; Chung *et al.*, 2004) was used to investigate the effect of losing *CaChitIV* and/or *CaPIK1* function on cell death-mediated defence signalling in pepper plants. Expression of *CaChitIV* and/or *CaPIK1* was significantly down-regulated during *Xcv* infection in pepper leaves in which *CaChitIV* and/or *CaPIK1* was silenced. Silencing either *CaChitIV* or *CaPIK1*, as well as both *CaChitIV* and *CaPIK1*, significantly enhanced *Xcv* growth but compromised the cell death response, ROS and NO accumulation, and defence response gene induction in pepper leaves during compatible and incompatible *Xcv* infections. Notably, co-silencing of both genes was much more effective at suppressing *CaPIK1-*triggered cell death, ROS and NO accumulation, and defence response gene induction than silencing either separately. It is concluded that *CaChitIV* expression enhances *CaPIK1-*triggered basal defence and HR-like cell death response in pepper leaves and is required for bacterial disease resistance. It has been proposed that recognition of pathogen-associated molecular patterns (PAMPs) by plant receptor-like kinase produces PAMP-triggered immunity (PTI) responses, including oxidative bursts, callose deposition, and defence gene induction (Zipfel *et al.*, 2004). It is suggested that, in pepper plants, activation of ROS bursts by the receptor-like cytoplasmic protein kinase, CaPIK1, acting together with the class IV chitinase, CaChitIV, triggers cell death and defence responses, resulting in reduced growth of *Xcv*. Silencing of *CaChitIV* alone did not compromise induction of the defence response genes *CaPR1* (PR1) (Kim and Hwang, 2000) and *CaDEF1* (defensin) (Do *et al.*, 2004), supporting the idea that *CaChitIV* expression assists *CaPIK1*-triggered cell death and defence responses. Moreover, *CaChitIV* expression may not positively regulate downstream defence genes in pepper.

The results provide the first evidence that CaChitIV specifically interacts with CaPIK1 in yeast and *in planta*. Moreover, it is shown that CaChitIV functions as an enhancer of *CaPIK1*-triggered cell death and defence responses. However, it remains to be clarified how secreted CaChitIV acts in the processes resulting in plant cell death and how signal transduction pathways triggered by *CaPIK1* regulate the activation of *CaChitIV* for plant defence.

## Supplementary data

Supplementary data are available at *JXB* online.

Figure S1. Nucleotide and predicted amino acid sequences of pepper *CaChitIV* cDNA.

Figure S2. (A) Comparison of the deduced amino acid sequence of CaChitIVwith sequences of class IV chitinases from tobacco, grapevine, *Arabidopsis*, rice, maize, and Norway spruce (B) A schematic diagram of domains in CaChitIV.

Figure S3. Subcellular localization of CaChitIV in onion epidermal cells following biolistic transformation

Figure S4. Quantitative real-time RT–PCR analysis of relative gene expression of *CaChitIV*, *CaPIK1*, *CaPR1*, and *CaDEF1* in pepper plants infected with virulent Ds1 (compatible) or avirulent Bv5-4a (incompatible) strains of *Xanthomonas campestris* pv. *vesicatoria*.

Figure S5. RT–PCR analysis of expression levels of *CaChitIV* in leaves from transgenic *Arabidopsis* empty vector control lines (00) and *CaChitIV*-OX lines #1, #2, and #3.

Table S1. Gene-specific primers for plasmid constructs used in this study.

Supplementary Data

## Abbreviations:

BiFC: bimolecular fluorescence complementation
CBD: chitin-binding domain
Co-IP: co-immunoprecipitation
DAB: 3,3′-diaminobenzidine
ER: endoplasmic reticulum
ETI: effector-triggered immunity
GlcNA: *N*-acetyl-d-glucosamine
HR: hypersensitive response
Hpa: *Hyaloperonospora arabidopsidis*
MAPK: mitogen-activated protein kinase
NO: nitric oxide
OX: overexpression
PCD: programmed cell death
PR: pathogenesis-related
R: resistance
RLCK: receptor-like cytoplasmic protein kinase
RLK: receptor-like kinase
ROS: reactive oxygen species
SAR: systemic acquired resistance
TRV: *Tobacco rattle virus*
VIGS: virus-induced gene silencing
Xcv: *Xanthomonas campestris* pv. *vesicatoria*.
