# Supplementary material for: The Capsicum annuum class IV chitinase ChitIV interacts with receptor-like cytoplasmic protein kinase PIK1 to accelerate PIK1-triggered cell death and defence responses
Source: J Exp Bot. 2015 Feb 17;66(7):1987–99. doi: 10.1093/jxb/erv001 (PMC4378632; doi:10.1093/jxb/erv001)
Supplement: Supplementary Data [file supp_66_7_1987__index.html]

The Capsicum annuum class IV chitinase ChitIV interacts with receptor-like cytoplasmic protein kinase PIK1 to accelerate PIK1-triggered cell death and defence responses — The Capsicum annuum class IV chitinase ChitIV interacts with receptor-like cytoplasmic protein kinase PIK1 to accelerate PIK1-triggered cell death and defence responses — Supplementary Data 

# The *Capsicum annuum* class IV chitinase ChitIV interacts with receptor-like cytoplasmic protein kinase PIK1 to accelerate PIK1-triggered cell death and defence responses

## Supplementary Data

Data files

**Files in this Data Supplement:**

- Supplementary Data - Supplementary Data
