# Supplementary material for: The Capsicum annuum class IV chitinase ChitIV interacts with receptor-like cytoplasmic protein kinase PIK1 to accelerate PIK1-triggered cell death and defence responses
Source: J Exp Bot. 2015 Feb 17;66(7):1987–99. doi: 10.1093/jxb/erv001 (PMC4378632; doi:10.1093/jxb/erv001)
Supplement: Supplementary Data [file supp_erv001_jexbot126938_file002.pdf]

The pepper class IV chitinase CaChitIV interacts with receptor-like cytoplasmic protein kinase CaPIK1 to accelerate CaPIK1-triggered cell death and defense responses

Dae Sung Kim, Nak Hyun Kim and Byung Kook Hwang

SUPPLEMENTARY DATA

```
                                                        aagatggg    8
cattaattctagtcatttaaaaaattcactgttggcctactggacacttaactaatcaag   68
ATGAACTTCTCTTCATCAACAAAATATTTCTTTCTCTTTGTAGCATTAGCTATAATAGCT  128
M  N  F  S  S  S  T  K  Y  F  F  L  F  V  A  L  A  I  I  A     20
GATGTACCAAGACTAATCTTGGCACAAAACTGTGGGTGTGCAGCAAATTTATGTTGTAGC  188
D  V  P  R  L  I  L  A  Q  N  C  G  C  A  A  N  L  C  C  S     40
AAATGGGGTTATTGTGGAGAGGGAAAGGATTATTGTGGTGAAGGGTGTCAAGGGGGGCCA  248
K  W  G  Y  C  G  E  G  K  D  Y  C  G  E  G  C  Q  G  G  P     60
TGTTTTAGTACTACACCATCAGGCAATAATGGCGGTTCAGTTTCTGATATTGTATCTGAT  308
C  F  S  T  T  P  S  G  N  N  G  G  S  V  S  D  I  V  S  D     80
GCATTCTTTAATGGGATAGTTGATCAAGCCGCTTCTAATTGTGAAGGAAAAGGGTTTTAT  368
A  F  F  N  G  I  V  D  Q  A  A  S  N  C  E  G  K  G  F  Y    100
TCAAGGGATAAATTCTTTGAAGCTCTTAAATCTTATCCTAACTTTGGAACTGTGGGTTCT  428
S  R  D  K  F  F  E  A  L  K  S  Y  P  N  F  G  T  V  G  S    120
AATGATGACTCTAAACGTGAAATTGCTGCTTTCTTTGCTCATGTCACCCACGAAACTGGC  488
N  D  D  S  K  R  E  I  A  A  F  F  A  H  V  T  H  E  T  G    140
CACATGTGCTACATAAATGAGATAAATGGTCCATCAGGCGACTATTGTGATGAGGACAAC  548
H  M  C  Y  I  N  E  I  N  G  P  S  G  D  Y  C  D  E  D  N    160
AAAGAGTACCCTTGTGTATCAGGCAAGAACTACTATGGTCGAGGACCAATTCAACTATCA  608
K  E  Y  P  C  V  S  G  K  N  Y  Y  G  R  G  P  I  Q  L  S    180
TGGAACTTCAACTACGGACCTGCTGGAAAATCCATTGGATTTGATGGCCTAAATGACCCT  668
W  N  F  N  Y  G  P  A  G  K  S  I  G  F  D  G  L  N  D  P    200
GACATAGTTGCAAGAGATGCTGTTATTTCCTTCAAGACAGCATTGTGGTATTGGATGAAC  728
D  I  V  A  R  D  A  V  I  S  F  K  T  A  L  W  Y  W  M  N    220
AATTGTCATTCACTAATTACTTCTGGACAAGGTTTTGGTCCAACTATTAGAGCTATTAAT  788
N  C  H  S  L  I  T  S  G  Q  G  F  G  P  T  I  R  A  I  N    240
GGTAGACTTGAATGTGATGGTGGTAATCCTCAAACTGTTGCTAGAAGGGTTGAGTATTAC  848
G  R  L  E  C  D  G  G  N  P  Q  T  V  A  R  R  V  E  Y  Y    260
ACTCAGTATTGTCAACAACTTGGTGTTGATGCTGGGGATAATCTCACGTGTTAGtttgca  908
T  Q  Y  C  Q  Q  L  G  V  D  A  G  D  N  L  T  C  *         277
agttagaataaacactatgattacactaagtcgctcacagactatacttatgagattata  968
cattcaaaaaaaaaaaaaaaa                                         990
```

Supplementary Fig. S1: Nucleotide and predicted amino acid sequences of pepper *CaChitIV* cDNA (accession no. KJ649334). The predicted amino acid sequence is given below the nucleotide sequence. The transcriptional start site is shown in bold type and the termination codon is marked with an asteri sk (*).

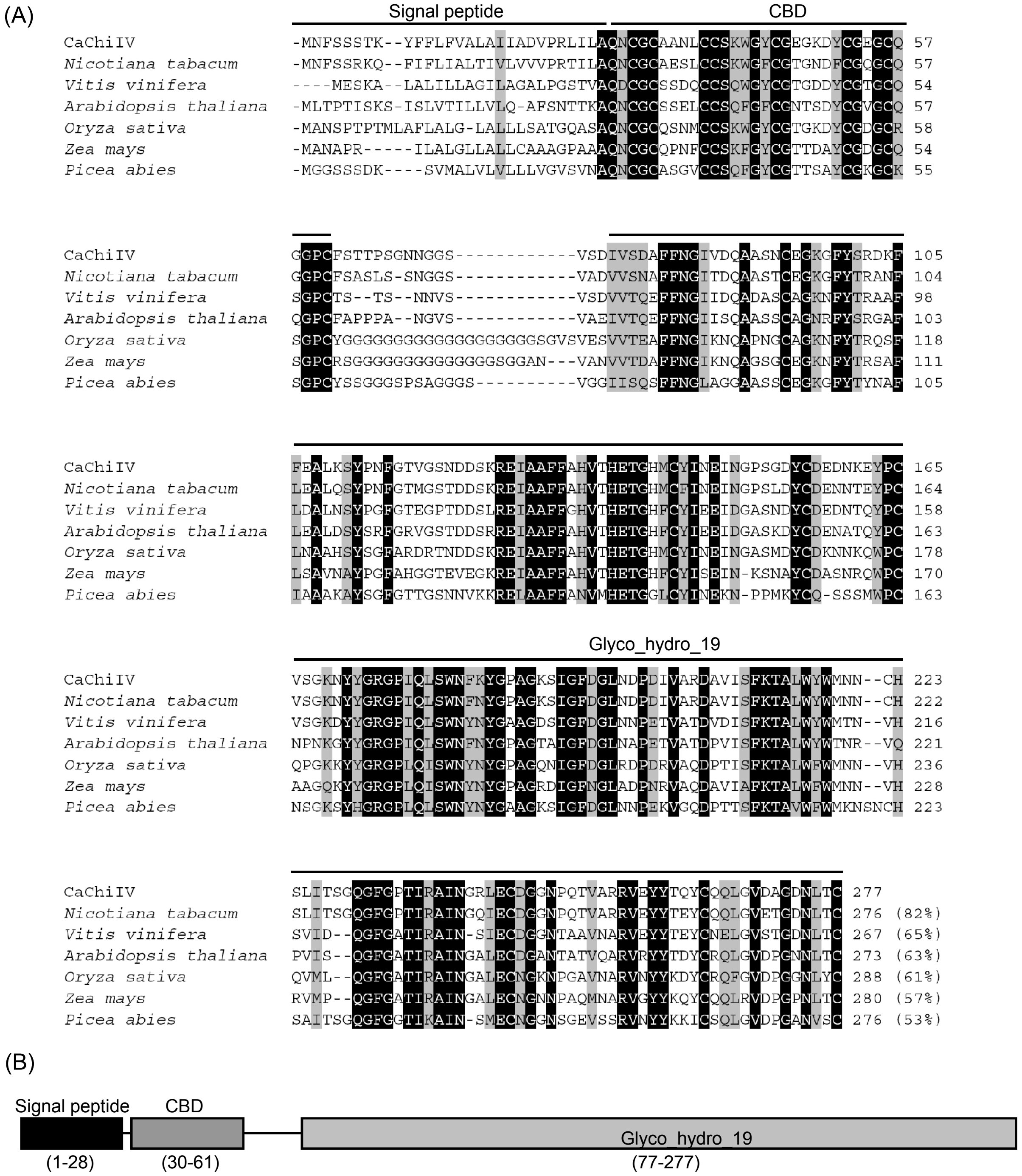

Supplementary Fig. S2: (A) Comparison of the deduced amino acid sequence of CaChitIVwith sequences of class IV chitinases from tobacco (*Nicotiana tabacum*; accession no. BAF44533), grape vine (*Vitis vinifera*; accession no. AAM95447), Arabidopsis (*Arabidopsis thaliana;* accession no. NP_191010), rice (*Oryza sativa;* accession no. NP_001053186), maize (*Zea mays*, accession no. NP_001158904) and Norway spruce (*Picea abies*, accession no. AY270017). Black or gray boxes indicate identical amino acids; dashes mark spaces inserted into the amino acid sequences for proper alignment. (B) A schematic diagram of domains in CaChitIV. Signal peptide: residues 1-28; chitin-binding domain (CBD): residues 30-61; glycoside hydrolase family 19 (glyco_hydro_19): residues 77-277.

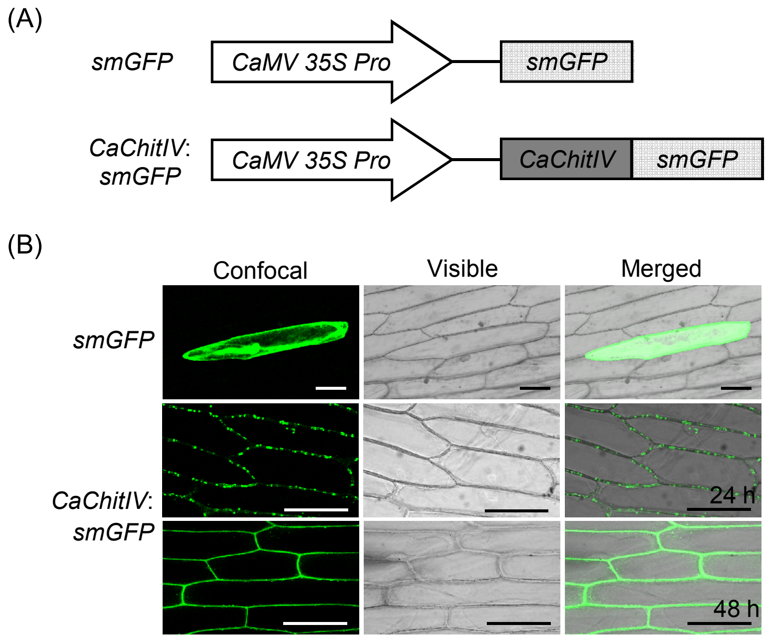

Supplementary Fig. S3: Subcellular localization of CaChitIV in onion epidermal cells following biolistic transformation. (A) Schematic diagram of the *smGFP*-tagged vector control and *CaChitIV* constructs. (B) Transient expression of *smGFP* and *CaChitIV*:*smGFP* in onion epidermal cells 24 h and 48 h after transformation, as detected by confocal laser-scanning microscopy. Scale bars: 100 μm.

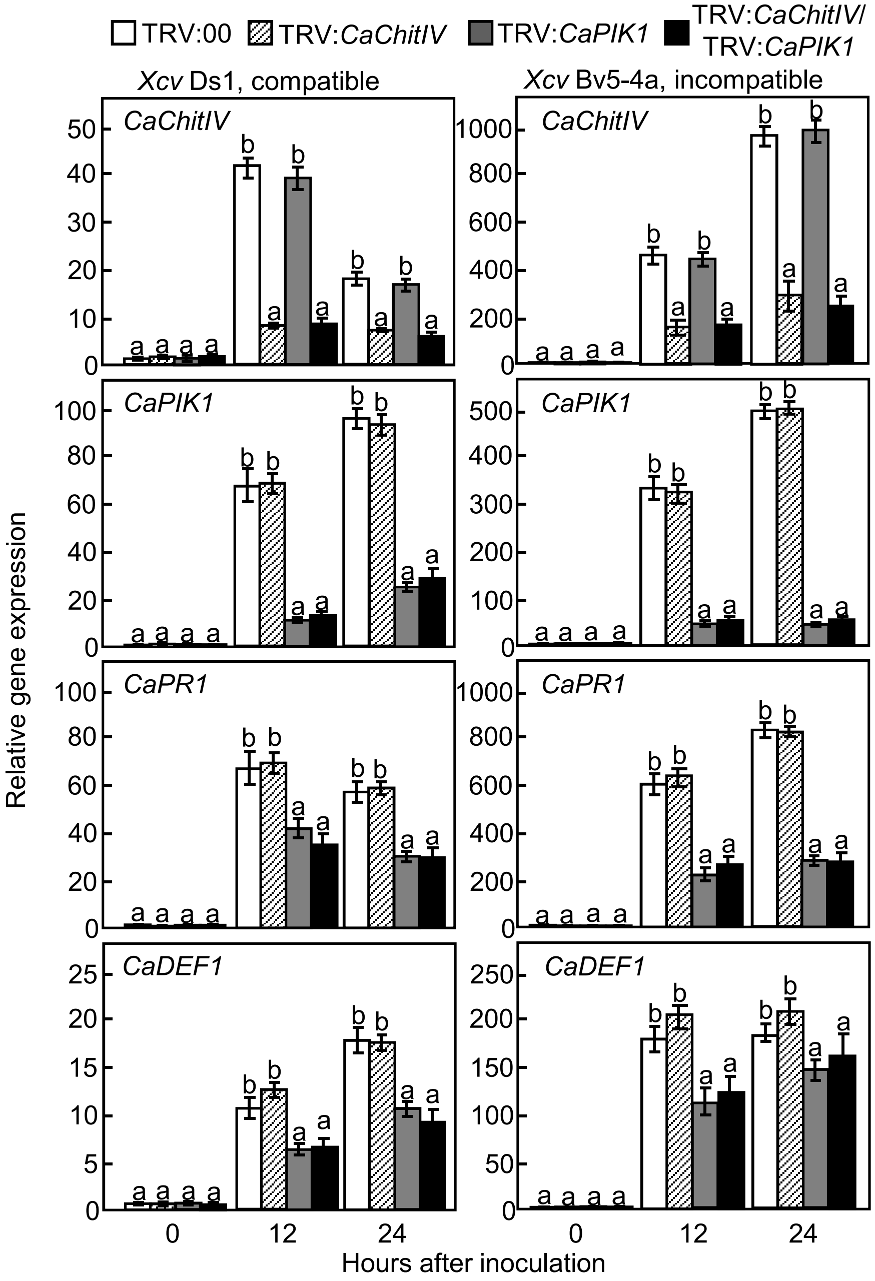

Supplementary Fig. S4: Quantitative real-time RT-PCR analysis of relative gene expre ssion of *CaChitIV*, *CaPIK1*, *CaPR1* and *CaDEF1* in pepper plants infected with virulent Ds1 (compatible) or avirulent Bv5-4a (incompatible) strains of *Xanthomonas campestris* pv. *vesicatoria* (*Xcv*). *CaPR1*: pathogenesis-related protein; *CaDEF1*: defensin. Express ion values were normalized by the expression levels of *Capsicum annuum18S rRNA.* D ata are the means ± standard deviations from three, independent experiments. Letters a bove the bars indicate statistically significant differences between treatments, according to the LSD test (*P*< 0.05).

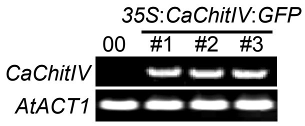

Supplementary Fig. S5: RT-PCR analysis of expression levels of *CaChitIV* in leaves from transgenic Arabidopsis empty vector control lines (00) and *CaChitIV*-OX lines #1, #2 and #3. *AtACT1* was used as an internal control.

Supplementary Table S1. Gene-specific primers for plasmid constructs used in this study

Analysis	Gene	Forward and Reverse Primer Sequences
Yeast two-hybrid	CaPIK1 F	5'-GAGAGAATTCATGGGGTGTTTCCCTTGTTCTG-3'
	CaPIK1 R	5'-GAGAGGATCCCCAGGAATCTTCATCGTTTATAGA-3'
	CaChitIV F	5'-GAGAGAATTCATGAACTTCTCTTCATCA-3'
	CaChitIV R	5'-GAGAGGATCCCTAACACGTGAGATTATC-3'
VIGS	CaPIK1 F	5'-CAAAACTGATAGTTTTGTAACTC-3'
	CaPIK1 R	5'-ACACTAATAAAAATTAATATACT-3'
	CaChitIV F	5'-AAGATGGGCATTAATTCTA-3'
	CaChitIV R	5'-CTTGATTAGTTAAGTGTCCAG-3'
BiFC	CaPIK1 F	5'-GAGATCTAGAATGGGGTGTTTCCCTTGTTCTG-3'
	CaPIK1 R	5'-GAGAGGATCCCCAGGAATCTTCATCGTTTATAGA-3'
	CaChitIV F	5'-GAGATCTAGAATGAACTTCTCTTCATCA-3'
	CaChitIV R	5'-GAGAGGATCCACACGTGAGATTATC-3'
pBIN35S and pBIN35S:GFP	CaChitIV F	5'-GAGATCTAGAATGAACTTCTCTTCATCA-3'
	CaChitIV R	5'-GAGAGGATCCACACGTGAGATTATC-3'
	CaChitIV ΔSP F	5'-GAGATCTAGAATGCAAAACTGTGGGTG-3'
	CaChitIV ΔSP R	5'-GAGAGGATCCACACGTGAGATTATC-3'
Quantitative real-time RT-PCR	CaChitIV F	5'-ATGAACTTCTCTTCATCAACAAAA-3'
	CaChitIV R	5'-CTAACACGTGAGATTATCCCCA-3'
	CaPIK1 F	5'-GGCTCTTGGTTCACTGGAAGATCATCTA-3'
	CaPIK1 R	5'-GCACAGTATCCATATGTACCCATCACTCTG-3'
	CaPR1 F	5'-CAGGATGCAACACTCTGGTGG-3'
	CaPR1 R	5'-CAAAGGCCGGTTGGTC-3'
	CaDEF1 F	5'-CAAGGGAGTATGTGCTAGTGAGAC-3'
	CaDEF1 R	5'-TGCACAGCACTATCATTGCATAC-3'
	Ca18S rRNA F	5'-AAACGGCTACCACATCCAAG-3'
	Ca18S rRNA R	5'-ACCCATCCCAAG GTTCAACT-3'
	CaACTIN F	5'-AAGCTCTCCTTTGTTGCTGTT-3'
	CaACTIN R	5'-GACTTCTGGGCATCTGAATCT-3'
Transgenic Arabidopsis	CaChitIV F	5'-GAGATCTAGAATGAACTTCTCTTCATCA-3'
	CaChitIV R	5'-GAGAGGATCCACACGTGAGATTATC-3'
	AtACT1 F	5'-CATCAGGAAGGACTTGTACGG-3'
	AtACT1 R	5'-GATGGACCTGACTCGTCAT-AC-3'
